# Halloysite Nanotubes Capturing Isotope Selective Atmospheric CO_2_

**DOI:** 10.1038/srep08711

**Published:** 2015-03-04

**Authors:** Subhra Jana, Sankar Das, Chiranjit Ghosh, Abhijit Maity, Manik Pradhan

**Affiliations:** 1 Department of Chemical, Biological & Macro-Molecular Sciences, S. N. Bose National Centre for Basic Sciences, Block - JD, Sector-III, Salt Lake, Kolkata - 700 098, India

## Abstract

With the aim to capture and subsequent selective trapping of CO_2_, a nanocomposite has been developed through selective modification of the outer surface of the halloysite nanotubes (HNTs) with an organosilane to make the nanocomposite a novel solid-phase adsorbent to adsorb CO_2_ from the atmosphere at standard ambient temperature and pressure. The preferential adsorption of three major abundant isotopes of CO_2_ (^12^C^16^O_2_, ^13^C^16^O_2_, and ^12^C^16^O^18^O) from the ambient air by amine functionalized HNTs has been explored using an optical cavity-enhanced integrated cavity output spectroscopy. CO_2_ adsorption/desorption cycling measurements demonstrate that the adsorbent can be regenerated at relatively low temperature and thus, recycled repeatedly to capture atmospheric CO_2_. The amine grafted halloysite shows excellent stability even in oxidative environments and has high efficacy of CO_2_ capture, introducing a new route to the adsorption of isotope selective atmospheric CO_2_.

The atmospheric concentration of CO_2_ which is currently ~390 ppm, has steadily been increasing since the beginning of the industrial revolution^1^,^2^. To meet our energy demands, the release of the greenhouse gas CO_2_ has exponentially increased day by day because of the growing dependence on fossil fuels. Under current projection, such an increase in CO_2_ concentration will lead to 500–1000 ppm by the year 2100. The growing concern about the consequences of higher concentrations of CO_2_ is not only the global warming but also the detrimental effect on the growth and physiology of the plants. Thus, the global attention has been focused on the efficient capture of CO_2_ from the atmosphere.

Amine based adsorbents have gained special interest for CO_2_ capture and sequestration as amines can selectively bind with CO_2_ even in presence of moisture at ambient temperature and pressure^3^,^4^,^5^. One of the most industrially established approaches for CO_2_ capture involved liquid-phase absorption using aqueous solution of amines^6^,^7^,^8^. The disadvantages of solvent based methods for CO_2_ capture are high energy requirement, degradation of amines during regeneration, contamination of the gas with solvent vapour, equipment corrosion, and environmental issues because of the loss of alkanolamine owing to its high volatile nature etc^8^,^9^. Therefore, there is a considerable interest to develop solid adsorbents because of their stability, reusability along with good CO_2_ sorption capacity. Another silent advantage of solid phase over solution phase adsorbent is the easy handling and simplified regeneration and separation procedure. Moreover, considerably low heat capacities of solid adsorbents may reduce the essential heat needed for the regeneration^4^,^10^. Recently, a variety of solid adsorbents have been developed to adsorb CO_2_, such as zeolites, porous silica, metal oxides, porous polymers, alumina, carbon, ion-exchange resin, nanofibrillated cellulose, metal organic frameworks^3^,^10^,^11^,^12^,^13^,^14^,^15^,^16^,^17^,^18^,^19^,^20^,^21^. However, most of the procedures require long time to reach saturation due to the slow adsorption kinetics, reduced activity in presence of moisture, sometimes low thermal and chemical stability.

The present work introduces the naturally occurring halloysite clay nanotubes (HNTs) as a solid adsorbent for CO_2_ capture due to their chemical tunability, high length-to-diameter ratio, and high temperature resistant property. Owing to their environmental friendly and biocompatible nature, much recent attention has turned towards HNTs for the development of a new inorganic-organic nanocomposite to explore in catalysis, drug delivery, as anticorrosion agents, adsorbent, and a nanoreactor to host reactants for nanosynthesis and biomimetic synthesis^22^,^23^,^24^,^25^,^26^,^27^,^28^,^29^. HNTs demonstrate positive as well as negative charge at their inner and outer surfaces respectively, as the internal surface composed of gibbsite-like array of Al-OH groups whereas the external surface consists of Si-O-Si groups^22^,^30^. Hence, we can tune the structure/properties of the HNTs through selective modification of the available negatively charged outer surfaces as well as positively charged inner surfaces. Despite the various applications of HNTs, there has been no report to our knowledge in the literature on the utilization of HNTs as an adsorbent to capture CO_2_ from the ambient air and the detailed study of the isotopically selective adsorption kinetics of CO_2_.

Herein, we first report the use of halloysite nanotube as a novel solid adsorbent to adsorb CO_2_ from the atmosphere, after the selective modification of the outer surface with (3-aminopropyl)triethoxysilane (Figure 1). Adsorption kinetics of CO_2_ on surface modified HNTs has been studied using a laser-based high-precision carbon dioxide isotope analyzer exploiting integrated cavity output spectroscopy technique. The preferential adsorption behavior of different isotopes of CO_2_ (^12^C^16^O_2_, ^13^C^16^O_2_, and ^12^C^16^O^18^O) by the present adsorbent has also been studied to obtain an insight into the efficacy of HNTs for atmospheric CO_2_ capture. Finally, we have demonstrated the efficient regenerability at relatively low temperature, recyclability, and stability of the adsorbent even in the oxidative environment; make the adsorbent to be an attractive one.

## Results

### Structural and morphological characterization of HNTs and functionalized HNTs

**Figure 2:**
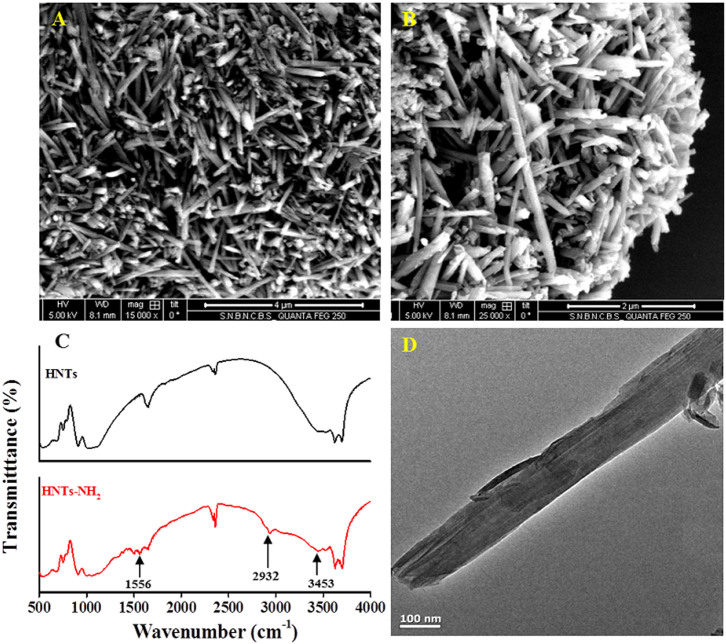
FESEM images of HNTs (A) before and (B) after surface modification with an aminosilane. (C) FTIR spectra demonstrate the chemical change due to the grafting of aminosilane on the surface of HNTs. (D) TEM image of aminosilane modified single HNT, representing HNTs consist of cylindrical shaped tubes with an open-ended lumen.

Halloysite nanotubes, having aluminium on the innermost and silicate on the outermost surfaces, allow different inner/outer surface chemistry. Since the surface defects generally takes place on the external surface of HNTs, the hydroxyl groups at those defects become accessible for surface modification^31^. Thus, selective surface modification of HNTs by (3-aminopropyl)triethoxysilane has been carried out to manipulate the chemico-physical properties through control chemistry of the constituents as well as morphology of these nanostructures. The chemical change due to the grafting of aminosilane on the surface of HNTs was analyzed by FTIR spectroscopy. Figure 2 represents the FTIR spectra of HNTs and amine functionalized HNTs (HNTs-NH_2_). The well-defined bands at 3621 and 3697 cm^−1^ in HNTs and HNTs-NH_2_ are assigned to the stretching vibrations of inner hydroxyl group and inner surface hydroxyl group respectively^31^. Three new peaks in HNTs-NH_2_ at 2932 and 3453 cm^−1^ are due to the stretching vibration of C–H and N–H and at 1556 cm^−1^ is assigned to the N-H deformation, signifying the grafting of APTES over the surface of HNTs. The diffraction pattern of HNT-NH_2_ is similar to that of bare HNTs, shown in Figure S1, Supplementary Information. The observed (020) reflection in both bare HNTs and HNTs-NH_2_ is the characteristic of tubular halloysite clay^32^,^33^. No intercalation of APTES into the interlayer of HNTs was confirmed as (001) reflection does not shift to the lower angles, indicating most of the hydroxyl groups are embedded because of the multi-layer structure of HNTs and thus, became unavailable for grafting. We have studied the thermal decomposition behavior of both HNTs and surface modified HNTs with the help of thermogravimetric analysis (TGA) in the temperature range 30–700°C under nitrogen flow to know the grafted amine/HNTs ratio. Desorption of physisorbed water from the surface of HNTs occurs around 50–150°C. The weight loss between 150–250°C, is due to the loss of hydrogen bonded aminosilane or removal of residual template^31^. Decomposition of grafted aminosilane over the surface of HNTs was observed around 250–475°C. The estimated mass loss for modified HNTs is consistent with the amount of aminosilane covalently bound to the HNTs (Figure S2 and Table S1, Supplementary Information). The weight loss above 375°C can be attributed to the dehydroxylation of the residual structural AlOH groups in both HNTs and HNTs-NH_2_.

**Figure 3:**
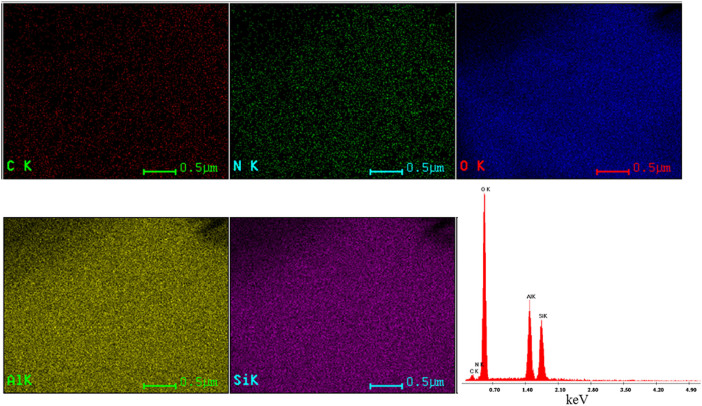
Element mapping by scanning electron microscopy and corresponding EDX spectrum of surface modified HNTs authenticated the presence of carbon and nitrogen with the three main constituents, oxygen, aluminium, and silicon in HNTs-NH_2_ due to the surface modification of HNTs by the aminosilane.

The morphology of HNTs before and after surface modification was characterized with the help of field emission scanning electron microscopy (FESEM). FESEM images of HNTs and HNTs-NH_2_ demonstrate that they consist of cylindrical shaped tubes with an open-ended lumen (Figure 2). The length of the tubes is 1.0 to 1.5 μm, having outer diameter of 50–100 nm and inner diameter of 15–20 nm. TEM image of HNTs–NH_2_ (Figure 2D) represents the cylindrical shaped tube composed of multilayer walls with the lumen. The observed defects on the surface of HNTs may be due to the mechanical damage or by crystallographic defects^23^,^31^. Energy dispersive X-ray analysis (EDX) further authenticated the presence of carbon and nitrogen with the three main constituents, oxygen, aluminium, and silicon in HNTs-NH_2_ (Figure 3) due to the surface modification of HNTs by APTES. In contrast, EDX spectrum of bare HNTs exhibits that they composed of aluminium, silicon, and oxygen only (Figure S3, Supplementary Information). Elemental quantification with mapping to know the basic composition of HNTs before and after surface modification has been carried out by SEM-EDX mapping. The elemental maps also demonstrate the presence of nitrogen in HNTs-NH_2_ (shown in Figure 3) which is achieved to be 0.58 wt%, representing the functionalization of HNTs by aminosilane. CHN elemental analysis further ascertains the accurate mass fraction of carbon, nitrogen, and hydrogen presented in the adsorbent. The fraction of C, N, and H in HNTs-NH_2_ are found to be 2.09, 0.53, and 2.18 wt% respectively. The estimated concentration of nitrogen in HNTs-NH_2_ indicates the immobilization of 0.38 mmol of amino groups on one gram of HNTs, which was also substantiated by TGA and EDX analysis. We have studied the entire experiment using this aminosilane modified HNTs as an adsorbent. However, we can tune the concentration of immobilized amino groups from 0.38 to 1.2 mmol g^−1^ of adsorbent.

### Atmospheric CO_2_ capture by functionalized halloysite nanotubes

**Figure 4:**
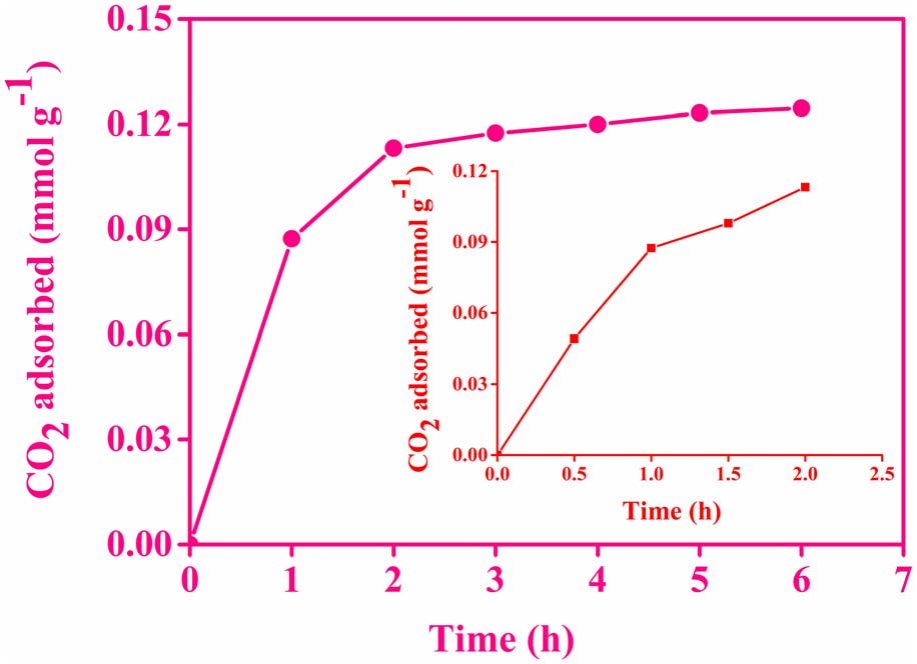
CO_2_ uptake from the ambient air by HNTs-NH_2_ as a function of time at 298 K. Inset shows CO_2_ uptake at an interval of 0.5 h up to 2 h. The plot represents a rapid adsorption phase followed by a slow approach to equilibrium.

To find out CO_2_ adsorption efficiency of aminosilane modified HNTs, ambient air was injected to the adsorbent bed and kept for a desired time to allow equilibration at room temperature (298 K) and pressure (1 atm). After the equilibration and adsorption, we have estimated the amount of unadsorbed CO_2_ remain in the ambient air of the flask by a laser-based ICOS technique. The amount of adsorbed CO_2_ was determined from the absolute change in concentration with respect to the blank flask. Figure 4 demonstrates time dependent adsorption of CO_2_ by HNTs-NH_2_ under dry condition. Amine functionalized HNTs exhibit maximum CO_2_ uptake efficiency, 2 h after the injection of ambient air and then it becomes levelling off. The steeper slope for the CO_2_ adsorption ascribed to the rapid rate of adsorption. With increasing adsorption time, the slope of the curve decreased and reached to an equilibrium. Hence, 2 h is the threshold time for this particular adsorbent to get saturated when the feed gas was ambient air.

We have further investigated the adsorption capacity of HNTs-NH_2_ at every 30 min up to the equilibrium time of 2 h, as we have observed a fast adsorption rate in this region. Inset of Figure 4 represents the CO_2_ uptake by HNTs-NH_2_ at an interval of 30 min. The plot shows a rapid adsorption phase followed by a slow approach to equilibrium. The fast adsorption kinetics of CO_2_ can be described to the presence of adequate active adsorption sites on the amine functionalized HNTs and enhanced adsorbent-adsorbate interaction. The slower adsorption rate may be attributed to the reduced available adsorption sites as well as increased diffusion resistance developed during CO_2_ adsorption^34^,^35^. However, bare HNTs hardly show any adsorption even after 12 h, shown in Figure S4, Supplementary Information.

**Figure 5:**
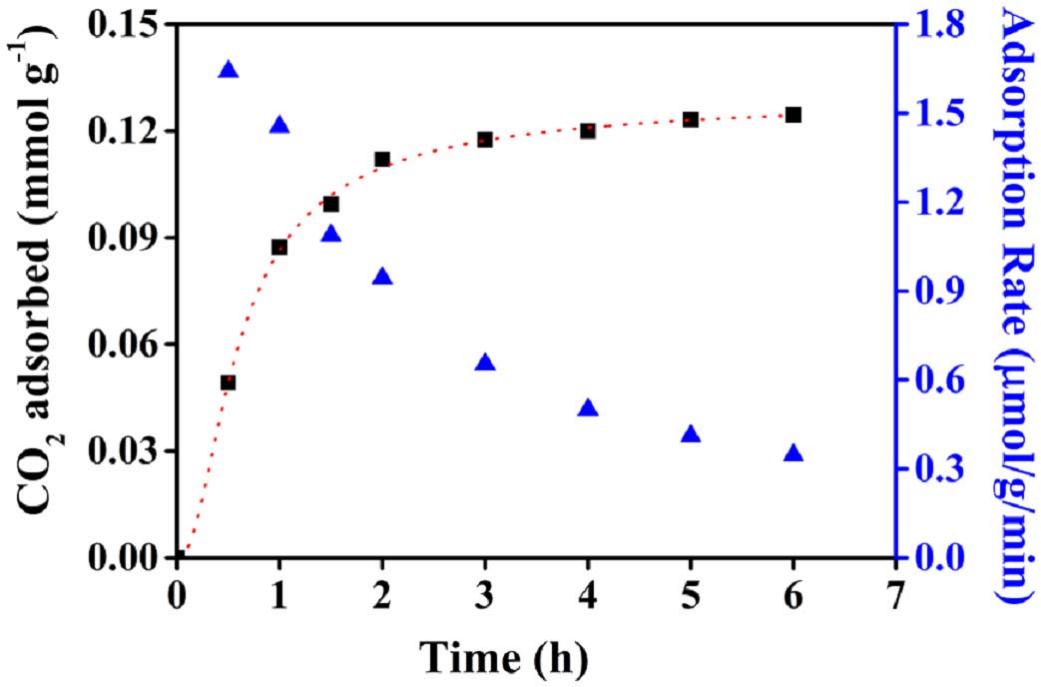
Fractional-order adsorption kinetics of CO_2_ from the ambient air by HNTs-NH_2_ and corresponding rate of CO_2_ adsorption.

The adsorption kinetics of CO_2_ on amine functionalized halloysite was studied considering pseudo-first-order^36^, pseudo-second-order^37^, and fractional-order rate equations respectively^38^.
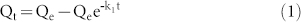

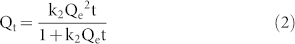

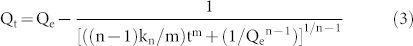
where, Q_e_ and Q_t_ are the adsorption capacity of the adsorbent at equilibrium and at time t. k_1_ and k_2_ are the rate constants for pseudo-first-order and pseudo-second-order reaction respectively. k_n_, m, and n are constants of this fractional-order model. The fractional-order kinetic model^38^ was recently proposed by Heydari-Gorji and Sayari to illustrate the rate of CO_2_ chemisorption on active sites of amine impregnated mesoporous silica where the adsorption rate is assumed to be directly proportional to the n^th^ power of the driving force and m^th^ power of the adsorption time. The value of n determines pseudo-order of the reaction with respect to driving force. As the model demonstrates the complexity of the reaction mechanism, k_n_ can be considered as an overall parameter combining several adsorption related factors^35^,^39^. Figure 5 represents the kinetic model on CO_2_ adsorption by HNTs-NH_2_ and corresponding rate of CO_2_ adsorption. The experimental data for CO_2_ adsorption by HNTs-NH_2_ was well fitted with fractional-order kinetic model (R^2^ = 0.999) than pseudo-first-order or pseudo-second-order model. The calculated characteristic parameters of the present model are k_n_ = 0.113 mmol^1−m^ g^m−1^ s^−n^, q_e_ = 0.13 mmol g^−1^, n = 2.564, and m = 2.018. Therefore, the adsorption kinetics of CO_2_ follows a general kinetic model based on fractional-order rate equation.

**Figure 6:**
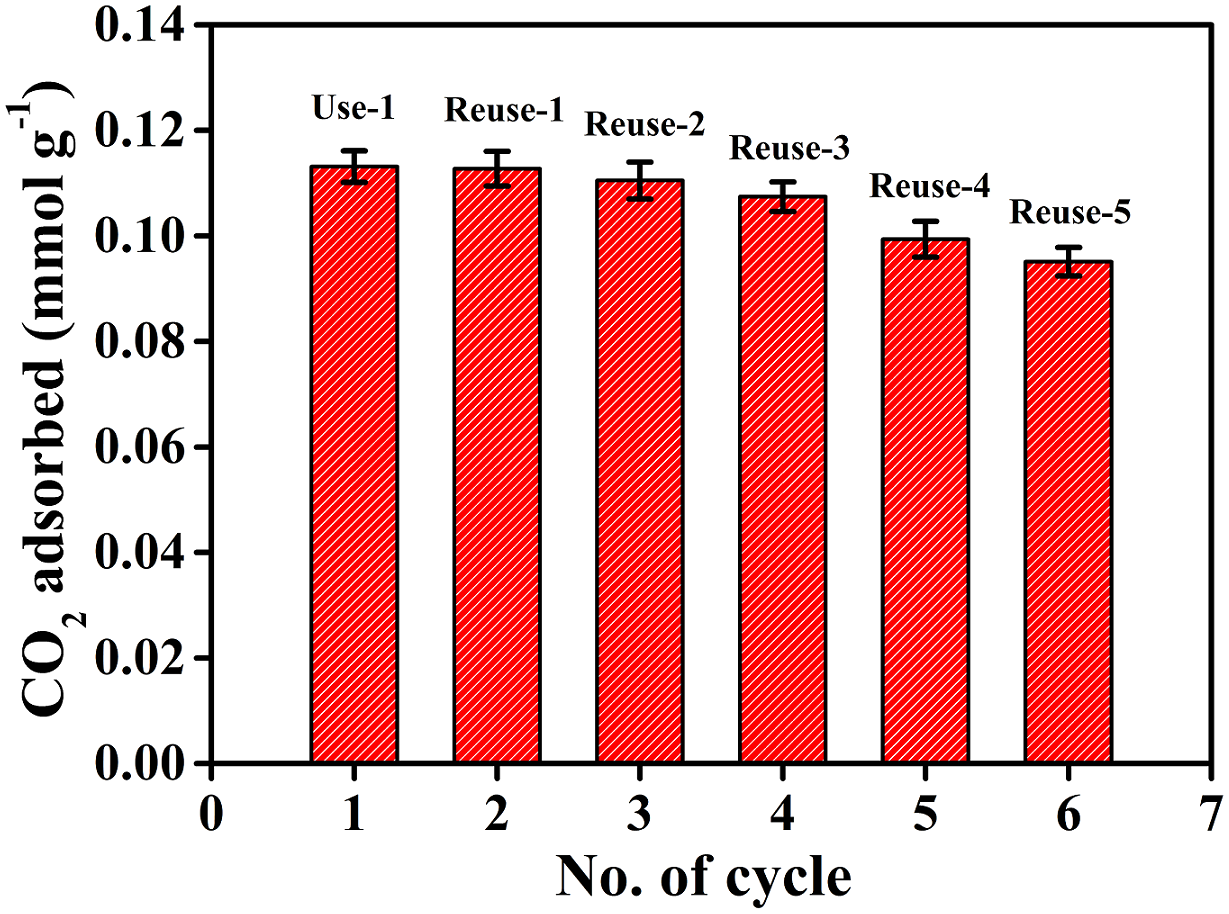
Multi-cycles adsorption of ambient CO_2_ using functionalized HNTs, demonstrating a stable adsorption/desorption cycling behavior of the adsorbent.

**Figure 7:**
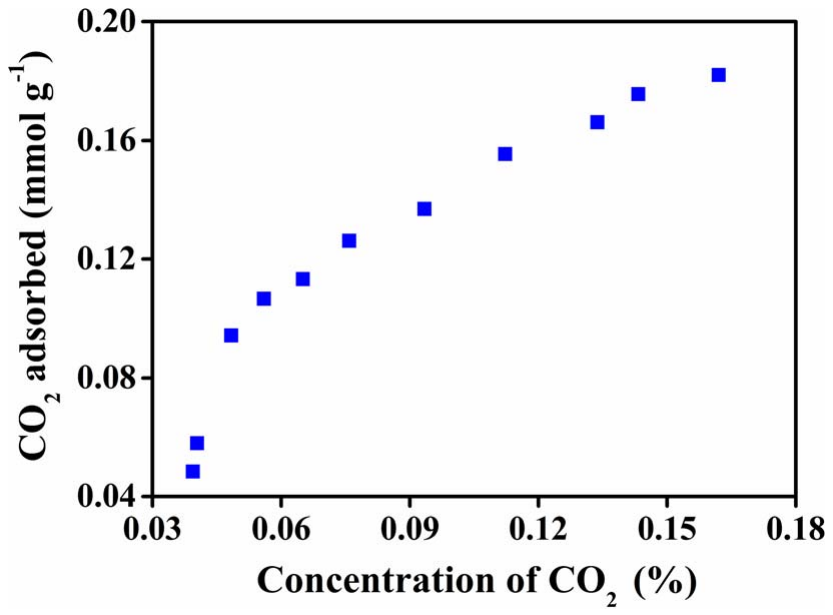
CO_2_ adsorption capacity of HNTs-NH_2_ as a function of CO_2_ concentration, representing concentration dependent CO_2_ adsorption by HNTs-NH_2_.

A stable adsorption/desorption cycling behavior of an adsorbent is desired to make the adsorbent robust and sustainable for practical usage. CO_2_ adsorption over a number of cycles using the same batch of modified HNTs has also been explored (Figure 6). To demonstrate the regenerability and recyclability of the adsorbent, consecutive 2 h adsorption/1.5 h desorption cycles were carried out. CO_2_ adsorption/desorption cycling experiments demonstrate that the surface modified HNTs can be regenerated upon heating at ~120°C under nitrogen flow followed by vacuum as mentioned earlier. Amine functionalized halloysite shows reversible CO_2_ adsorption behavior and thus after regeneration, the adsorbent was recycled a couple of times to capture CO_2_. Since desorption started readily at low temperature, the compounds formed between CO_2_ and the amine are not stable enough and thus release CO_2_ under reduced pressure. After prolonged cyclic experiment, HNT-NH_2_ exhibits a slightly decreasing trend in the adsorption efficiency. We have varied the concentration of CO_2_ keeping the other experimental condition the same. Figure 7 represents concentration dependent CO_2_ adsorption by HNTs-NH_2_ in dry condition. After 2 h of equilibrium, the amount of CO_2_ adsorbed by the adsorbent was estimated. Amine functionalized halloysite demonstrates strong dependence on the CO_2_ feed concentration.

## Discussion

The efficacy of an amine based adsorbent was determined based on the maximum amine efficiency i.e.; the ratio of the moles of CO_2_ adsorbed to the moles of amine present in an adsorbent. The adsorption capacity of HNTs-NH_2_ for CO_2_ capture from ambient air has been found to be 0.13 mmol g^−1^ of adsorbent. The amine efficiency of HNT-NH_2_ has been calculated to be 33% when the ambient air was used as feed gas. The estimated concentration of -NH_2_ and CO_2_ represents the presence of sufficient amounts of active adsorption sites in the modified halloysite and the -NH_2_ groups are embedded on the external surfaces of HNTs. Additionally, the above result also established that the adsorption performance of amine grafted halloysite entirely depends on the surface density of the amine groups which are covalently attached to the surface. Efficiency of an amine based adsorbent also depends on the type of amine used as well as reaction condition whether it is humid or dry. In absence of moisture, carbamates were formed due to the reaction between R-NH_2_ and CO_2_ through a zwitterionic mechanism^40^,^41^. Carbamate formation can be expressed as follows:
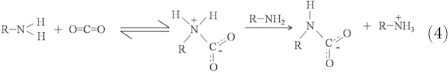
According to M. Caplow, the formation of carbamate involved the neucleophilic attack of the lone pair on nitrogen of R-NH_2_ to CO_2_ to produce zwitterions, followed by the deprotonation of those zwitterions by a base to form carbamates^40^. In our study, another amine will act as the free base as we have carried out this experiment under anhydrous condition. FTIR spectrum recorded after the adsorption of CO_2_ on HNTs-NH_2_ also represents the presence of alkylammonium carbamate species on aminosilane modified HNTs (see Figure S5 and Table S2, Supplementary Information).

To investigate whether the ambient CO_2_ adsorption by amine functionalized halloysite is isotopically selective; we have studied the adsorption trend of all the three major abundant isotopes of CO_2_. It is worth noting that the ambient air contains 0.04% of carbon dioxide having isotopic abundances of ^12^C^16^O_2_ (98.42%), ^13^C^16^O_2_ (1.1%) and ^12^C^16^O^18^O (0.394%). The ratio of isotopic data for ^13^CO_2_ and ^12^C^16^O^18^O in our study were expressed by the conventional notation, δ^13^C and δ^18^O in per mil (‰) relative to the standard Pee Dee Belemnite (PDB), which can be described as the following:
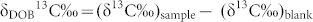

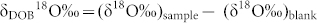

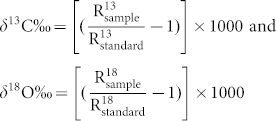

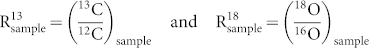
where, 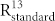 and 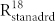 are the international standard Pee Dee Belemnite (PDB) values i.e. 0.0112372 and 0.0020672 respectively.

**Figure 8:**
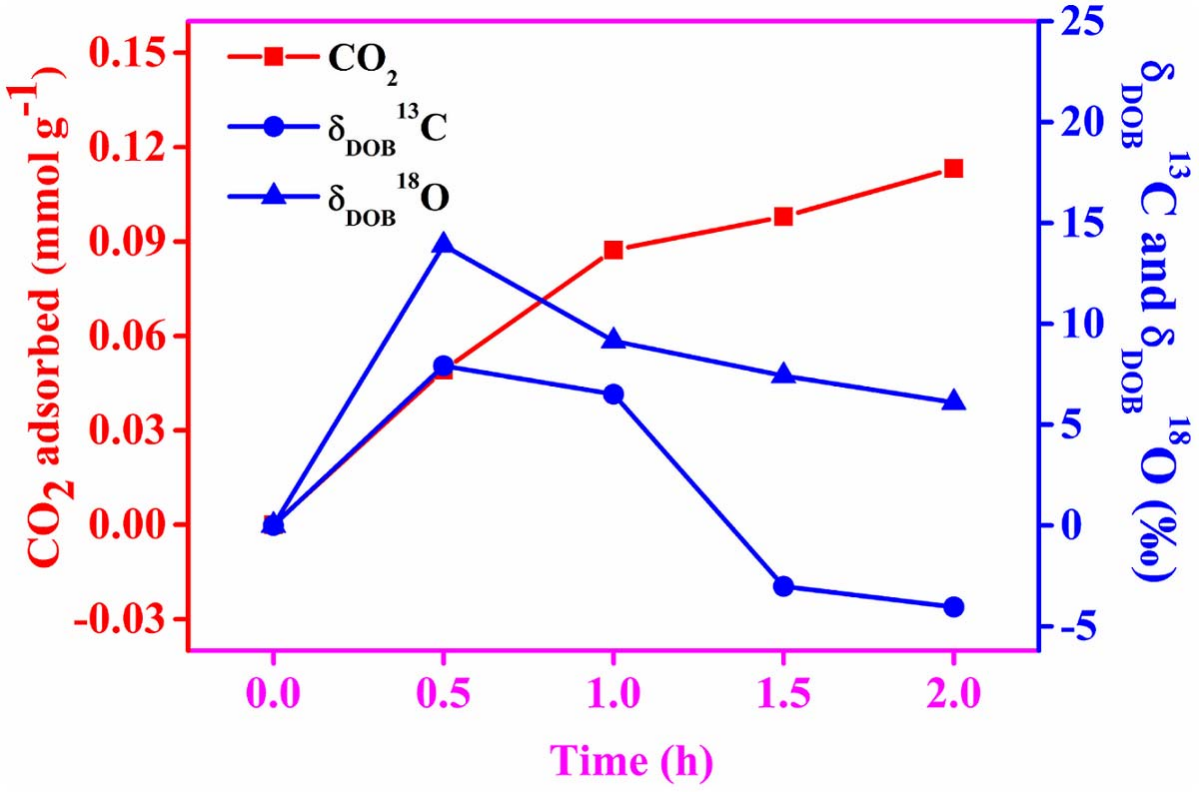
Adsorption kinetics of major abundant isotopes of CO_2_ present in ambient air. The adsorption of ^13^CO_2_ and ^18^O of CO_2_ are expressed as δ_DOB_^13^C‰ and δ_DOB_^18^O‰.

**Figure 9:**
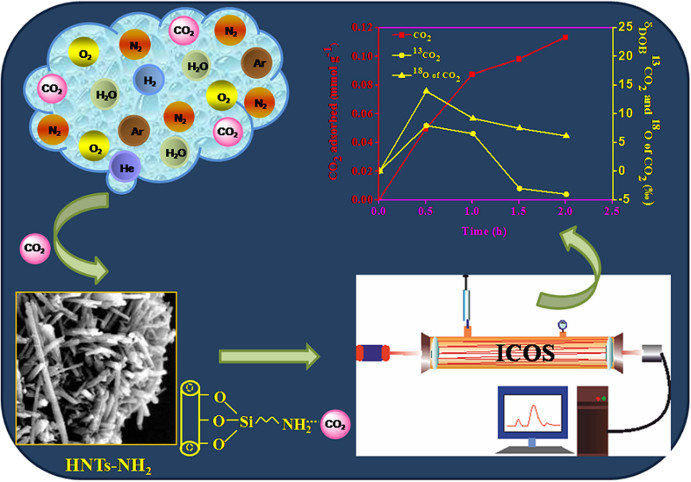
Preferential adsorption of three major abundant isotopes of atmospheric CO_2_ (^12^C^16^O_2_, ^13^C^16^O_2_ and ^12^C^16^O^18^O) by amine functionalized halloysite nanotubes has been demonstrated using a laser based high-resolution carbon dioxide analyzer based on the integrated cavity output spectroscopy technique.

The adsorption kinetics of the three major abundant isotopes of CO_2_ is shown in Figure 8. We have observed an increase in isotope ratio for both the isotopomers of CO_2_ (^13^CO_2_ and ^12^C^16^O^18^O; which are basically expressed here as δ^13^C and δ^18^O of CO_2_) into the unadsorbed CO_2_ present in the ambient air of sealed sample flask in the early stage (up to 0.5 h) of adsorption. As the chemical behavior of an atom completely depends on its electronic structure, isotopes of an element demonstrate almost identical chemical behavior. Therefore, we would expect same adsorption rate of the three different isotopes of CO_2_. The enhancement of ^13^CO_2_ and ^18^O of CO_2_ value in the sample flask compared to blank (expressed as δ_DOB_^13^C and δ_DOB_^18^O of CO_2_) was found to be maximum at 0.5 h, after which the observed value began to drop steadily until it came into near the equilibrium after 1.5 h of the adsorption. The above observation from the present kinetic study suggests a preferential adsorption of ^12^CO_2_ on amine functionalized nanotubes at the very beginning rather than ^13^CO_2_ and ^18^O of CO_2_, resulting an added accumulation of later two isotopes of CO_2_ into the ambient air of the sample flask. This may be due to the relatively highest abundance of ^12^CO_2_ molecules in the injected air compared to the other isotopomers of CO_2_ to be adsorbed by the adsorbent and thus, may increase the isotope ratio of (^13^C/^12^C)_sample_ and (^18^O/^16^O)_sample_. Once the sample flask was allowed to keep at room temperature for more time to reach equilibrium, the other isotopes of CO_2_ (^13^CO_2_ and ^12^C^16^O^18^O) come near the vicinity of the adsorbent and results in a subsequent increase in the adsorption rate of ^13^CO_2_ and ^12^C^16^O^18^O on amine modified halloysite. The gradual increase in adsorption rate up to 1.5 h indicates that this preferential isotopic adsorption may tend to come into equilibrium as the time progresses and finally it becomes almost levelling off beyond 2 h of adsorption. The adsorption kinetics of the isotopes of atmospheric CO_2_ up to 6 h has been presented in Figure S6, Supplementary Information. Thus, laser-based high-resolution isotopic CO_2_ analyzer based on integrated cavity output spectroscopy holds a great promise as a tool to investigate the preferential adsorption of different isotopes of CO_2_ with time by means of diverse adsorbents as depicted in Figure 9.

In summary, we have synthesized amine grafted HNTs which demonstrate reversible CO_2_ capture activity from the ambient air. The adsorption of atmospheric CO_2_ by this adsorbent follows fractional-order kinetic model. The time-dependent adsorption behavior of different isotopes of CO_2_ present in the ambient air has also been studied. High efficacy of CO_2_ capture, easy regeneration and reuse, excellent stability of the surface modified halloysite, make the procedure more robust, environmentally friendly, and sustainable. Furthermore, this procedure also represents an additional advantage of solid-phase over solution-phase adsorbent, as solvent loss and corrosion issues resulting from the use of aqueous amines would be minimized in case of solid adsorbent and thus, surface modified halloysite becomes a promising candidate for CO_2_ capture. Nevertheless, our findings shed light on the preferential isotopic CO_2_ adsorption which may open a new route in the frontier area of CO_2_ capture and sequestering study even under ultra-dilute condition.

## Methods

### Surface modification of halloysite nanotubes

Surface modification of halloysite nanoclay was carried out under nitrogen atmosphere using standard air-free techniques^42^. A 50 mL round bottom flask was fitted with a condenser, rubber septum, thermocouple adaptor, and quartz sheath in which a thermocouple was inserted. Then, 1 g of HNTs was taken in that flask containing 12 mL of toluene and the reaction mixture was heated with a heating mantle. At 60°C, 1.0 mL of APTES was injected to the flask and then the temperature of the reaction mixture was increased to 120°C and refluxed at that temperature for 12 h. Once the flask was cooled down to room temperature, the product was washed several times with toluene and ethanol respectively and then dried at 100°C under vacuum. After the surface modification, APTES functionalized HNTs was abbreviated as HNTs-NH_2_.

### Adsorption studies

For all adsorption experiments, we have collected the ambient air and used that as feed gas to study the CO_2_ adsorption capacity of the surface modified HNTs. CO_2_ adsorption experiments were carried out at ambient pressure and temperature using amine grafted HNTs as an adsorbent in a round bottom flask, which was fitted with a rubber septum and two adapters. The adsorbent was pre-treated with ultra-high pure nitrogen gas at 120°C for a period of 1 h and then pull down vacuum for another 45 min before adsorption to ensure complete desorption of pre-adsorbed CO_2_ if any. Keeping the same experimental condition, another round bottom flask without any adsorbent was also fitted with a rubber septum and the adapters. After cooling to room temperature, ambient air was injected to the adsorbent bed of the sample flask and the blank flask followed by equilibration for a desired time. After the equilibration, we have measured the amount of unadsorbed CO_2_ remain in the ambient air of the flask. CO_2_ uptake was monitored by a laser-based ICOS technique. The amount of adsorption of CO_2_ was determined from absolute change in concentration with respect to blank flask. Relative humidity has been observed within 30–32% during the experiment.

### CO_2_ adsorption measurement by ICOS

We have utilized a laser-based high-resolution carbon dioxide analyzer based on integrated cavity output spectroscopy (ICOS), to measure the amount of unadsorbed CO_2_ and its isotopic composition. ICOS is basically a cavity-enhanced laser-absorption technique to analyze the stable isotope ratios of carbon dioxide (^12^C^16^O^16^O, ^13^C^16^O^16^O and ^12^C^16^O^18^O) in real time with a typical precession of ±0.25‰. The technical details of ICOS spectrometer (CCIA 36-EP, Los Gatos research, USA) have been described elsewhere^43^. In brief, the ICOS spectrometer is comprised of a continuous wave diode laser operating at ~2.05 μm and a high-finesse optical cavity. The two high reflectivity mirrors (R ~ 99.98%) at the two ends of the measurement cell (~59 cm) allow the laser light to move back and forth provide an effective optical path-length of ~3 km^44^. The laser frequency was tuned to scan over 20 GHz to record the absorption spectra of ^12^C^18^O^16^O (4874.178 cm^−1^), ^12^C^16^O^16^O (4874.448 cm^−1^) and ^13^C^16^O^16^O (4874.086 cm^−1^) in the (2,0^0^,1)←(0,0^0^,0) vibrational combination band of the CO_2_ molecule. The temperature of the cavity was maintained at 46°C by a resistive heater, whereas the pressure within the cavity was regulated by a diaphragm pump.

## Author Contributions

S.J. conceived the idea and designed the experiment. S.D., C.G. and A.M. performed the experiments. S.J. and M.P. contributed to the results analysis and discussion. S.J. wrote the paper and managed the overall execution of the project.

## Supplementary Material

Supplementary Information Supplementary Information

## Figures and Tables

**Figure 1:**
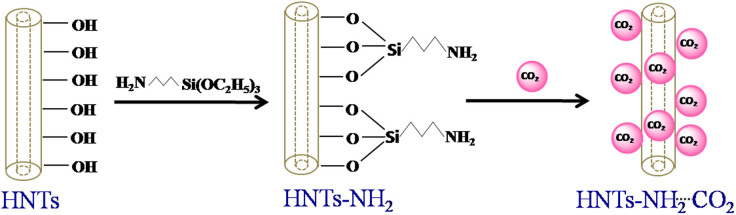
Schematic presentation of the surface modification of HNTs with (3-aminopropyl) triethoxysilane, followed by atmospheric CO_2_ capture at standard ambient temperature and pressure.
