# Supplementary material for: Halloysite Nanotubes Capturing Isotope Selective Atmospheric CO2
Source: Sci Rep. 2015 Mar 4;5:8711. doi: 10.1038/srep08711 (PMC4348623; doi:10.1038/srep08711)
Supplement: Supplementary Information [file srep08711-s1.pdf]

Supplementary Information

Halloysite Nanotubes Capturing Isotope Selective Atmospheric CO_2

Subhra Jana[1], Sankar Das[1], Chiranjit Ghosh[1], Abhijit Maity[1] & Manik Pradhan[1]

[1]Department of Chemical, Biological & Macro-Molecular Sciences, S. N. Bose National Centre for Basic Sciences, Block - JD, Sector-III, Salt Lake, Kolkata - 700 098, India.

Correspondence and requests for materials should be addressed to S.J.
(subhra.jana@bose.res.in)

Experimental Section

Materials

All chemicals were used as received without any further purification. Halloysite nanotubes (HNTs) and (3-aminopropyl) triethoxysilane (APTES, 97%) were purchased from Sigma-Aldrich and Alfa Aesar respectively. Toluene and ethanol were received from Merck, India.

Materials Characterization

Powder X-ray diffraction (XRD) data were recorded on a PANalytical X-PERT PRO powder diffractometer using Cu Kα radiation with 45 kV beam voltage and 40 mA beam current. Thermogravimetric analyses (TG) were performed on a Perkin Elmer DAIMOND TG/DTA instruments. Fourier transform infrared (FTIR) spectra were collected using JASCO FTIR 6300. CHN analysis was done by PerkinElmer 2400 Series II CHNS Elemental Analyzer. The morphology of the halloysite nanotubes was characterized using field emission scanning electron microscope (FESEM: FEI QUANTA FEG 250). Elemental mapping and energy dispersive X-ray analysis (EDX) have been carried out in the above mentioned FESEM.

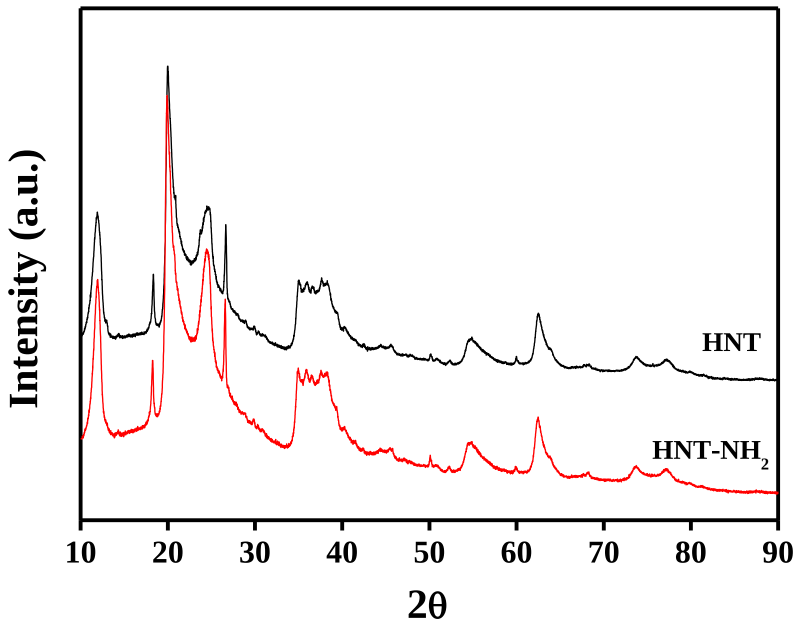

Figure S1. XRD patterns of HNTs before and after surface modification using (3-aminopropyl) triethoxysilane.

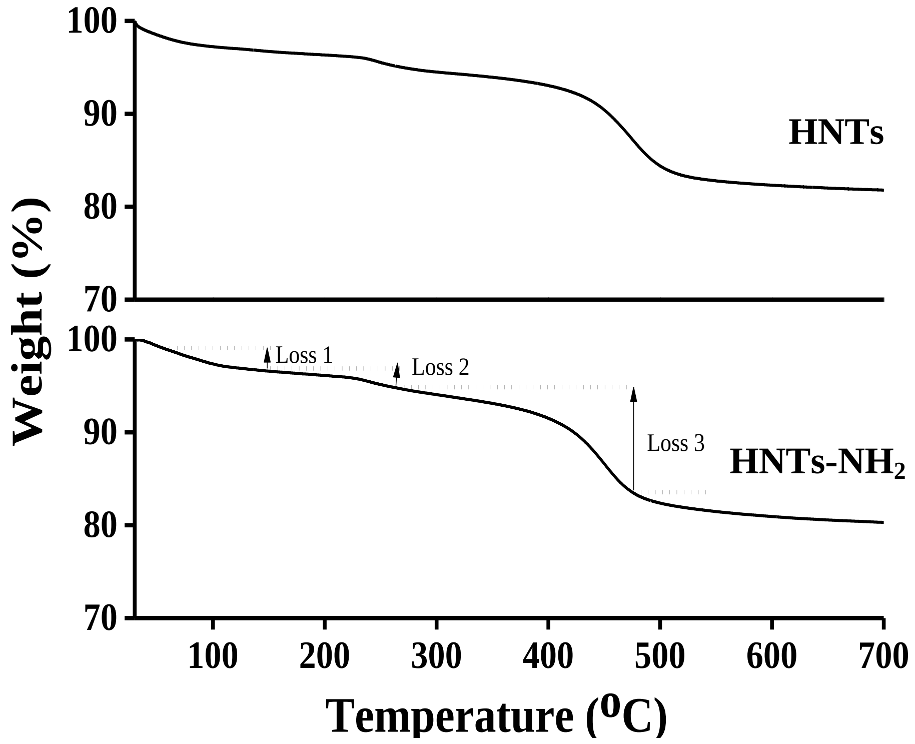

Figure S2. Thermogravimetric analysis (TGA) data of HNTs and surface modified HNTs measured under nitrogen from 30 to 700°C. The amount of APTES ligand covalently bound to the HNTs was estimated to be ~ 3.4 wt%.

Table S1: Mass losses of aminosilane modified halloysite nanotubes during TGA analysis

**Temperature Range (°C)**	**Mass Loss**	**Assignments**
50-150	Loss 1	Desorption of physisorbed water from the surface.
150-250	Loss 2	Loss of hydrogen bonded aminosilane or removal of residual template.
250-475	Loss 3	Decomposition of grafted aminosilane over the surface of HNTs and dehydroxylation of the residual structural AlOH groups.

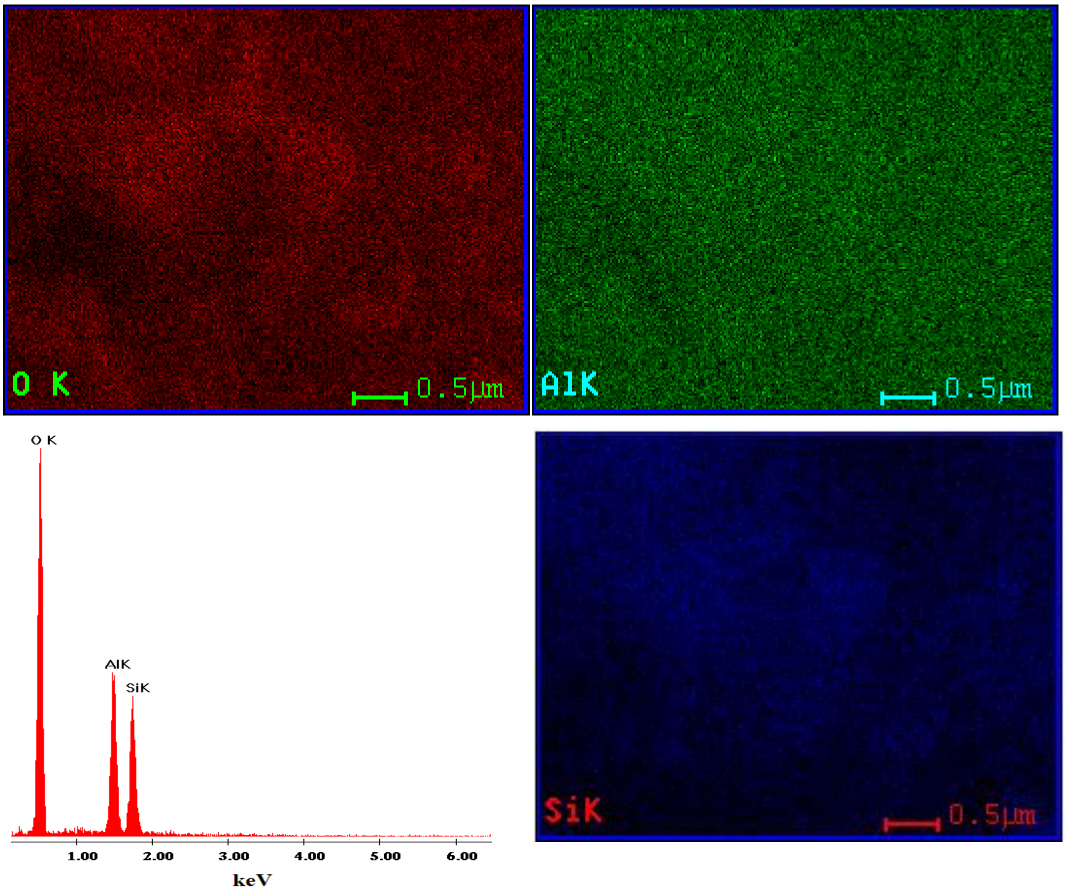

Figure S3. Element mapping by scanning electron microscopy and corresponding EDX spectrum of bare HNTs.

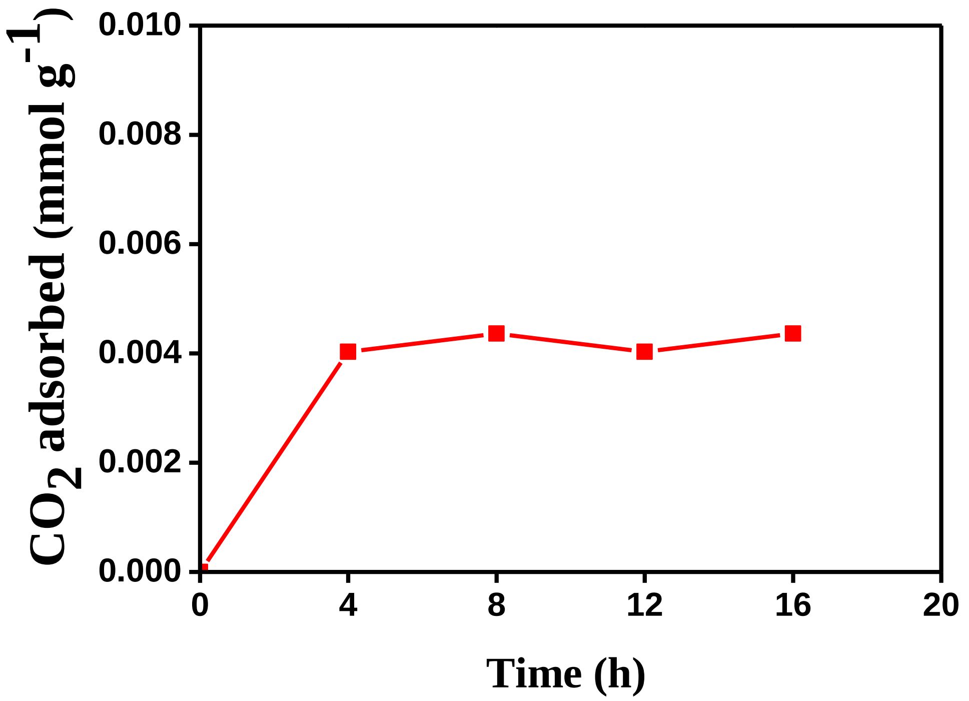

Figure S4. CO_2 uptake from ambient air by bare HNTs as a function of time at 298 K. The adsorption capacity of atmospheric CO_2 by bare HNTs was studied up to 16 h and it was found that the bare HNTs show a very minimum adsorption of CO_2 (~ 3%) compared to HNTs-NH_2. The minimum adsorption of CO_2 by bare HNTs is because of their hollow structure, as HNTs were evacuated for a period before CO_2 adsorption study.

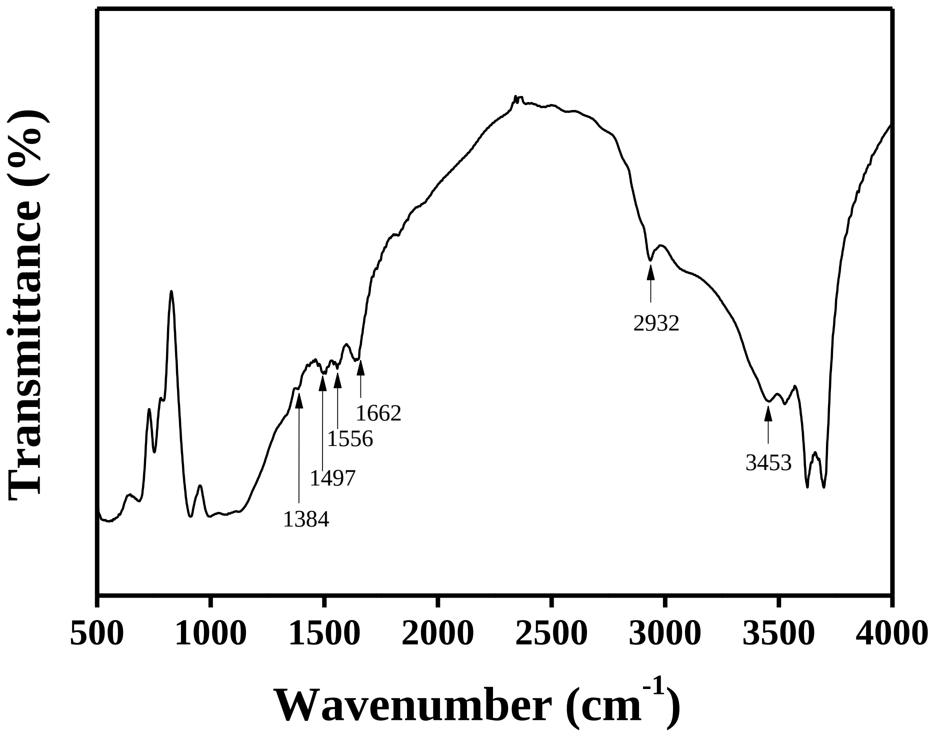

Figure S5. FTIR spectrum of CO_2 adsorbed on aminosilane modified HNTs (HNTs-NH_2).

Table S2: Position and assignment of FTIR bands

**Wavenumber (cm^{-1})**	**Assignment**	**Species**	**Reference**
1384	Symmetric stretching COO-	Carbamate	1, 2
1497	Symmetric NH^+ deformation	Ionic carbamate	3
1556	N-H deformation	APTES	4
1662	C=O stretching	Carbamic acid	1, 5
2932	C–H stretching vibration	APTES	3, 4
3453	N–H stretching vibration	APTES	4

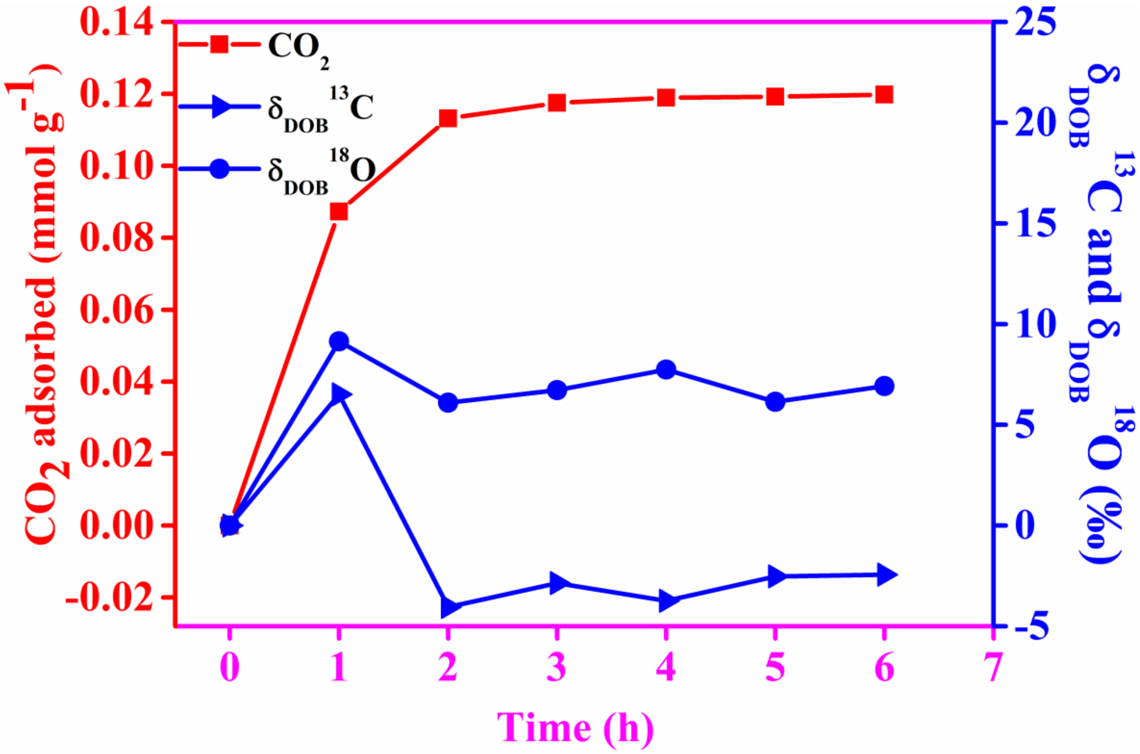

Figure S6. Adsorption kinetics of major abundant isotopes of CO_2 present in ambient air. The adsorption of $^{13}CO_2$ and ^{18}O of CO_2 are expressed as $\delta_{DOB}{}^{13}C$‰ and $\delta_{DOB}{}^{18}O$‰.

References

1. Knöfel, C., Martin, C., Hornebecq, V. & Llewellyn, P. L. Study of Carbon Dioxide Adsorption on Mesoporous Aminopropylsilane-Functionalized Silica and Titania Combining Microcalorimetry and in Situ Infrared Spectroscopy. *J. Phys. Chem. C* **113**, 21726–21734 (2009).
2. Alkhabbaz, M. A., Bollini, P., Foo, G. S., Sievers, C. & Jones, C. W. Important Roles of Enthalpic and Entropic Contributions to CO2 Capture from Simulated Flue Gas and Ambient Air Using Mesoporous Silica Grafted Amines. *J. Am. Chem. Soc.* **136**, 13170−13173 (2014).
3. Zhao, A., Samanta, A., Sarkar, P. & Gupta, R. Carbon dioxide adsorption on amine-impregnated mesoporous SBA-15 sorbents: experimental and kinetics study. *Ind. Eng. Chem. Res.* **52**, 6480−6491 (2013).
4. Yuan, P. *et al.* Functionalization of halloysite clay nanotubes by grafting with γ-aminopropyltriethoxysilane. *J. Phys. Chem. C* **112**, 15742-15751 (2008).
5. Bacsik, Z., Ahlsten, N., Ziadi, A., Zhao, G., Garcia-Bennett, A. E., Martín-Matute, B. & Hedin, N. Mechanisms and Kinetics for Sorption of CO_2 on Bicontinuous Mesoporous Silica Modified with *n*-Propylamine. *Langmuir* **27**, 11118–11128 (2011).
